# Structural and Potential Functional Properties of Alkali-Extracted Dietary Fiber From *Antrodia camphorata*

**DOI:** 10.3389/fmicb.2022.921164

**Published:** 2022-07-07

**Authors:** Yongjun Xia, Peng Meng, Shaodong Liu, Zhuoming Tan, Xi Yang, Lihong Liang, Fan Xie, Hui Zhang, Guangqiang Wang, Zhiqiang Xiong, Jenyu Lo, Lianzhong Ai

**Affiliations:** ^1^School of Health Science and Engineering, Shanghai Engineering Research Center of Food Microbiology, University of Shanghai for Science and Technology, Shanghai, China; ^2^Honest and Humble Biotechnology Co., Ltd., New Taipei City, China

**Keywords:** *Antrodia camphorata*, alkali-extracted dietary fibers, adsorption capacities, immunomodulatory activities, structure

## Abstract

*Antrodia camphorata* is rich in a variety of bioactive ingredients; however, the utilization efficiency of the residue of *A. camphorata* is low, resulting in serious waste. It is necessary to deeply study the functional components of *A. camphorata* residues to achieve high-value utilization. In this study, the components, structural characteristics, and functional properties of alkali-extracted dietary fiber extracted from residues of *A. camphorata* (basswood and dish cultured fruiting body, respectively) were investigated. There were similar components and structural characteristics of ACA-DK (extract from basswood cultured) and ACA-DF (extract from dish cultured). The two alkali-extracted dietary fiber were composed of mainly cellulose and xylan. However, ACA-DK has better adsorption capacities than ACA-DF on lipophilic substances such as oil (12.09 g/g), cholesterol (20.99 mg/g), and bile salts (69.68 mg/g). *In vitro* immunomodulatory assays stated that ACA-DK had a good effect on promoting the proliferation of RAW 264.7 cells and can activate cell phagocytosis, NO synthesis, and other immune capabilities. The edible fungus *A. camphorata* is a good source of functional dietary fiber. The alkali-extracted dietary fiber of *A. camphorata* might be used as a functional ingredient in the medicine and food industry.

## Graphical Abstract

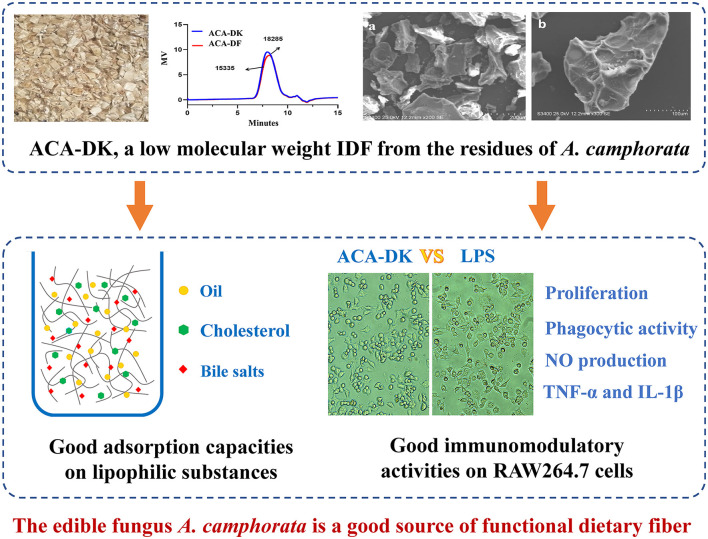

## Introduction

Dietary fiber plays an important role in the inhibition of disease occurrence and development (Chawla and Patil, 2010). High dietary fiber intake could significantly reduce the morbidity and mortality of coronary heart disease, stroke, type 2 diabetes, and colorectal cancer (Marlett et al., 2002). The physical and chemical properties of dietary fiber such as solubility, viscosity, and fermentability determine its function in the gastrointestinal tract, including the influence of nutrient utilization, intestinal motility, stool formation, and microbial specificity (Rodriguez et al., 2020; Gill et al., 2021; Ma et al., 2021). Dietary fiber has a good effect on improving digestive function and lipid metabolism disorders. Bo et al. (2021) have shown that dietary fiber derived from bean dregs has good adsorption ability *in vitro*. However, the functions of dietary fiber from different sources are quite different. Dietary fiber extracted from grain has a protective effect on T2DM, while fruit fiber has almost no protective effect (Davison and Temple, 2018).

*Antrodia camphorata* is a unique fungus species with good nutritional and medicinal values (Lu et al., 2013; Kuang et al., 2021). It contains many biological active substances, including polysaccharides, triterpenes, superoxide dismutase, and sterols (Chien et al., 2008; Lin et al., 2017; Zhang et al., 2018). Studies have shown that dietary fiber is an important active component of *A. camphorata*, especially water-soluble dietary fiber (polysaccharides). Macrophage RAW 264.7 is a good *in vitro* experimental platform to measure the immunomodulatory or the anti-inflammatory activity of polysaccharides (Shen et al., 2017). Chen et al. (2007) isolated five water-soluble polysaccharide components from *A. camphorata* mycelium, and the results showed that the component AC-2 had a significant anti-inflammatory effect on LPS-induced RAW 264.7 cells. In addition, the polysaccharides from extracts of *A. camphorata* mycelia or fruiting bodies show the function of antiangiogenic, anti-inflammatory, and immunomodulatory activities (Cheng et al., 2008, 2016; Meng et al., 2012). However, few studies report the immunomodulatory function of water-insoluble dietary fiber from *A. camphorata* in RAW 264.7 cells.

The active ingredients in the fruiting bodies of *A. camphorata*, such as polysaccharides and triterpenes, are prepared into extracts or dripping pills after leaching with ethanol and hot water. However, a large number of residues after leaching have not been well used, and there is almost no research on alkali-extracted dietary fiber in residues of *A. camphorata*. The extraction residue of edible fungi still contains more functional components, which could be used in functional foods (Kao et al., 2012). Edible fungi residue contains a large amount of dietary fiber, which is also a major source of dietary fiber in addition to grains, fruits, and vegetables. A series of dietary fiber obtained from fruit bodies of mushroom have the functions of adsorbing heavy metals and regulating immunity and anti-hyperlipidemia (Hua et al., 2012; Ren et al., 2017; Choma et al., 2018). In this study, two kinds of residues of *A. camphorata* fruit body are used as the raw materials to analyze the structural characteristics of dietary fiber. Then, the *in vitro* adsorption and immunomodulatory activity of dietary fiber from different sources were studied. This research lays the foundation for further application of *A. camphorata* residues.

## Materials and Methods

### Materials

In this experiment, the residues of *A. camphorata* fruit body were the gifts from Honest and Humble Biotechnology Co., Ltd. (New Taipei City, China) and stored in the refrigerator at 4°C. The residues were extraction by hot water (95°C for 2 h) before experiments, to remove water-soluble polysaccharides. All the monosaccharide standards, MTT, lipopolysaccharide (LPS), and bovine serum albumin (BSA) were purchased from Sigma-Aldrich Co. (Shanghai, China). High-glucose DMEM was purchased from Wisten Biotechnology Co. Ltd. (Nanjing, China). A mouse IL-6 ELISA kit (Catalog number: M6000B) and a TNF-α ELISA kit (Catalog number: MTA00B) were purchased from R&D Systems China Co. Ltd. (Shanghai, China). All other reagents used in this study were of analytical grade.

### Pretreatment of AC Residues

*Antrodia camphorata* residues were washed with ethanol and distilled water. Then, the residues were dried at 60°C and ground to a powder (filtered by a 60-mesh sieve). The powder was packaged and stored at −20°C until further analysis.

### Extraction of Dietary Fiber

The dietary fiber were extracted from *A. camphorata* residues using the described method with some modifications (Wang et al., 2013). Briefly, the powder of *A. camphorata* residues was extracted by 0.01 M NaBH_4_/0.5 M NaOH solution in the solid-to-liquid ratio of 1:10 for 12 h at 4°C. After filtration, the extraction solution was neutralized by glacial acetic acid, placed at 4°C for 4 h, and then centrifugated at 8,000 rpm for 15 min at 4°C, and then the solid residues were collected. The solid residues were washed with distilled water three times and resuspended in the DMSO (contain 0.25 M LiCl), followed by centrifugated at 8,000 rpm for 15 min at 4°C. The supernatants were dialyzed for 3 d and freeze drying at −80°C for 72 h. The dietary fiber extracted from the fruiting body of *A. camphorata* was coded as ACA-DK. The dietary fiber extracted from dish-cultured hypha of *A. camphorata* was coded as ACA-DF.

### Determination of Molecular Weight and Component Analysis

The molecular weights of dietary fiber were determined by using an HPLC system (Waters 1525 system, Agilent PLgel MIXED column 5 μm), with DMSO as the mobile phase at a flow rate of 1 mL/min at 35°C. Molecular weights of dietary fiber were calculated by the retention time.

Total sugars were estimated by the phenol–sulfuric acid method (DuBois et al., 1956) using an Evolution 300 UV spectrophotometer (Evolution 300, Thermo Fisher Scientific, England). The concentration of uronic acid was evaluated by using the m-hydroxydiphenyl method (Blumenkrantz and Asboe-Hansen, 1973). The moisture contents of dietary fiber were determined by using a moisture analyzer (MB45, OHAUS). The content of protein was determined using the Kjeldahl method with a nitrogen-to-protein conversion factor of 6.25 (AOAC 955.04, AOAC, 2000). The content of dietary fiber was determined by the method described by Yeh et al. (2005).

### Scanning Electron Microscopy

Morphological characterization of ACA-DK and ACA-DF was performed according to the method described by Ullah et al. (2017) with some modifications. The samples were fixed on double-sided conducting scotch tapes and coated with a 10-nm gold layer. Subsequently, the surface microstructure of samples was observed using a scanning electron microscope (Hitachi S-4800, Hitachi, Ltd., Tokyo, Japan) at a voltage of 25 kV.

### Monosaccharide Compositions

Monosaccharide composition of dietary fiber were measured by HPAEC-PAD (Dionex ICS 3000) (Yang et al., 2009). Briefly, 100 uL of ACA-DK and ACA-DF (5 mg/mL) were put into the hydrolysis tube, respectively, and then hydrolyzed with 4 mol/L trifluoroacetic acid (TFA) for 2 h at 110°C. The hydrolysates were dried with nitrogen in a water bath at 65°C. Then the hydrolysates were dissolved in water and filtered with a 0.22-μm microporous membrane. HPAEC-PAD was performed to determine the monosaccharide composition with a CarboPacTM PA20 column (6.5 μm, 3.0 × 150 mm i.d.) and eluted with a gradient mobile phase at a flow rate of 0.5 mL/min. The injection volume of samples was 20 μL. The mobile phase consisted of A: H_2_O, B: NaOH (20 mmol/L) and C: NaOAc (1 mol/L). The elution condition started A:B:C = 92:8:0; 0-20 min, linear gradient to A:B:C =87:8:5; 20-30 min, linear gradient to A:B:C = 78:8:20; 30-50 min, linear gradient to A:B:C = 20:80:0.

### Fourier-Transformed Infrared Spectroscopy

The dietary fiber samples (1 mg) were well ground and blended with KBr (9 mg) and then pressed into a 1 mm pellet for FT-IR analysis (IRAfinity-1S, Shimadzu) in the resolution range from 400 to 4,000 cm^−1^.

### X-Ray Diffraction

Crystalline structural analysis of the freeze-dried samples was performed by using an X-ray diffractometer (MiniFlex600) with a Cu-Kα radiation source (λ = 1.542 Å) at a voltage of 40 kV and an incident current of 40 mA (González et al., 2011). The diffraction intensities were scanned from 4 to 70° with an angle step width of 0.02°. The crystallinity index (CrI) of the dietary fiber samples was calculated from the peak area under the curve by MDI Jade 6.5 software; the equation is as follows (1):


(1) $$\begin{array}{l} Crl=\frac{I_{002}-I_{am}}{I_{002}} \end{array}$$

where CrI is the relative degree of crystallinity, I_002_ is the maximum intensity of the crystalline diffraction peak which is located at a diffraction at 2θ = 22.13°, and I_am_ is the lowest intensity of diffraction at 2θ = 18°-19°, while I_am_ represents the amorphous part.

### Water Holding Capacity and Water Swelling Capacity

WHC was measured according to the methods reported by Luo et al. (2018). The samples (1.0 g) were hydrated in 20 mL deionized water at room temperature for 18 h. The samples were centrifuged at 4,000 rpm for 15 min and weighted immediately. The WHC was calculated by Equation (2):


(2) $$\begin{array}{l} WHC\left(\text{g}/\text{g}\right)=\frac{m_{2}-m_{1}}{m_{1}} \end{array}$$

where m_2_ is the weight of the residue (g) containing water and m_1_ is the weight of the dry sample (g).

WSC was determined by the method reported by Sowbhagya et al. (2007) with appropriate modifications. The samples (0.5 g) were hydrated in 20 mL distilled water in a graduated tube for 18 h at room temperature to swell fully for swelling volume detection. The bed volumes of mixtures were recorded. WSC was calculated by the following Equation (3):


(3) $$\begin{array}{l} WSC\left(\text{mL}/\text{g}\right)=\frac{V_{1}-V_{0}}{M_{0}} \end{array}$$

where V_2_ is the volume of the hydrated sample, V_0_ is the volume of the sample prior to hydration, and M_0_ is the weight of the sample prior to hydration.

### Oil Adsorption Capacity

Oil adsorption capacity was measured according to the method of Luo et al. (2018). The sample (1.0 g) was added to 20 mL soybean oil in a 50-mL centrifuge tube. After mixing for 30 s, the mixture was incubated at 25°C for 18 h. Then the tubes were centrifuged at 4,000 rpm for 15 min to remove the supernatant (excess oil), and the residue was weight. Oil adsorption capacity (OAC) was expressed as the amount of absorbed oil per gram of the sample (g oil / g dry weight). The OAC was calculated using Equation (4):


(4) $$\begin{array}{l} OAC\left(g/g\right)=\frac{M_{3}-M_{1}}{M_{1}} \end{array}$$

where M_3_ is the weight of the residue (g) containing oil and M_1_ is the weight of the dry sample (g).

### Cholesterol Binding Capacity

Cholesterol binding capacity of the samples were investigated according to the procedure described by previously reported with slight modification (Luo et al., 2018). A fresh egg yolk was diluted with 9 times of its weight distilled water and then homogenized by stirring. The samples (0.5 g) were mixed with 50 mL diluted yolk solution in a centrifuge tube at pH 2.0 and 7.0 (simulating the pH conditions in the stomach and small intestine, respectively). The mixture was placed in an oscillating incubator at 37°C for 2 h and then centrifuged at 4,000 rpm for 20 min at room temperature. The supernatant was diluted with acetic acid and mixed with o-phthalaldehyde at room temperature for 20 min. Then, the absorbance of the supernatant was measured at 550 nm. The amount of cholesterol was quantified based on a standard curve, and CAC was calculated according to Equation (5):


(5) $$\begin{array}{l} CBC\left(\text{mg}/\text{g}\right)=\frac{C_{1}-C_{2}}{M_{0}} \end{array}$$

where C_1_ is the weight of cholesterol before adsorption, C_2_ is the weight of cholesterol after adsorption, and M_0_ is the weight of the sample.

### Sodium Cholate Binding Capacity

The sodium cholate binding capacity of dietary fiber was determined according the method of Luo et al. (2018) with some modification. Samples (0.125 g) were incubated with 0.15 mmol/L sodium cholate in 100 mL of sodium phosphate buffer (pH 7.0). The slurry was shaken at 120 rpm for 2 h in a 250-mL flask maintained at 37°C and then centrifuged at 5,000 rpm for 20 min, and the unbinding cholate in the supernatant was determined at 620 nm with a spectrophotometer (UV-2600, Island ferry, Japan). The binding capacity of sodium cholate (SCBC) was calculated by Equation (6):


(6) $$\begin{array}{l} SCBS(\text{mg/g})=\frac{S_{1}-S_{2}}{S_{0}} \end{array}$$

where S_1_ is the content of sodium cholate in the solution before adsorption, S_2_ is the remaining content of sodium cholate in the supernatant after adsorption, and S_0_ is the mass of the sample taken.

### RAW 264.7 Cell Proliferation Assay and Morphology Observation

Cell proliferation was quantified by a standard MTT assay (Zheng et al., 2019). RAW 264.7 cells (1 × 10^5^ cells/well, DEME per well) were seeded in 96-well culture plates under conditions (5% CO_2_, 37°C) for 24 h. The DEME mediums containing different concentrations of ACA-DK and ACA-DF (10, 50, 100, 200, 500 μg/mL) were added into each well and incubated with the cells for 24 h. LPS (1 μg/mL) was used as the positive control group. After incubation, the medium was removed, and cells were incubated with 100 μL of MTT (0.5 mg/mL) solutions for 4 h. Next, the MTT solution was discarded, and dimethyl sulfoxide (100 μL of DMSO) was added and shaken for 10 min until no visible particulate matter detected. Finally, the absorbance was measured at 570 nm, and the cell proliferation ratio was determined. The morphology of the RAW 264.7 cells was observed *via* an inverted fluorescence microscope.

### Phagocytic Activity Assay in RAW 264.7 Cells

The promoting effects of ACA-DK and ACA-DF on phagocytosis of RAW 264.7 cells were determined by neutral red uptake assay (Liu et al., 2017). RAW 264.7 cells (1 × 10^5^ cells/well, DEME per well) were seeded in 96-well culture plates under conditions (5% CO_2_, 37°C) for 24 h. The DEME medium containing different concentrations of ACA-DK and ACA-DF (10, 50, 100, 200, 500 μg/mL) was added into each well and incubated with the cells for 24 h. LPS (1 μg/mL) was used as the positive control group. After incubation, the medium was removed, and the cells were incubated with neutral red (v/v, 0.075%) solutions for 30 min. Next, the supernatant was discarded, and the cells were washed with PBS (200 μL per well) for three times at room temperature to remove the redundant neutral red. Then, a 100 μL of the lysis solution contained an equal volume ratio of ethanol (v/v, 50%), and acetic acid (v/v, 1%) was added into the well and lysed at 4°C for 12 h. Finally, the absorbance was measured at 540 nm, and the phagocytic activity was determined.

### Measurement of NO Production in RAW 264.7 Cells

RAW 264.7 cells (1 × 10^5^ cells/well, DEME per well) were seeded in 96-well culture plates under conditions (5% CO_2_, 37°C) for 24 h. The RAW 264.7 cells were treated for 24 h with different concentrations of ACA-DK and ACA-DF (10, 50, 100, 200, 500 μg/mL). LPS (1 μg/mL) was used as the positive control group. After incubation, the supernatant was collected from cultures and mixed with an equal volume of Griess reagent and incubated for 30 min at room temperature. Nitrite production was determined by measuring absorbance at 540 nm with NaNO_2_ as a standard.

### Determination of TNF-α and IL-1β Production in RAW 264.7 Cells

RAW 264.7 cells (1 × 10^5^ cells/well, DEME per well) were seeded in 96-well culture plates under conditions (5% CO_2_, 37°C) for 24 h. The RAW 264.7 cells were treated for 24 h with different concentration of ACA-DK and ACA-DF (10, 50, 100, 200, 500 μg/mL). LPS (1 μg/mL) was used as the positive control group. After incubation, the supernatant was collected from cultures for analysis of TNF-α and IL-1β using enzyme-linked immunosorbent assay (ELISA) kits according to the manufacturer's instructions.

### Statistical Analysis

All data are expressed as means ± standard deviation for the in dependently performed experiments. One-way factorial analysis of variance (ANOVA) was used to assess the differences between mean values. Statistical analysis was performed with ANOVA (SPSS 24.0). A *P*-value of < 0.05 or 0.01 was considered statistically significant.

## Results

### Chemical Composition, Monosaccharide Composition, and Molecular Weight of ACA-DK and ACA-DF

**Figure 1:**
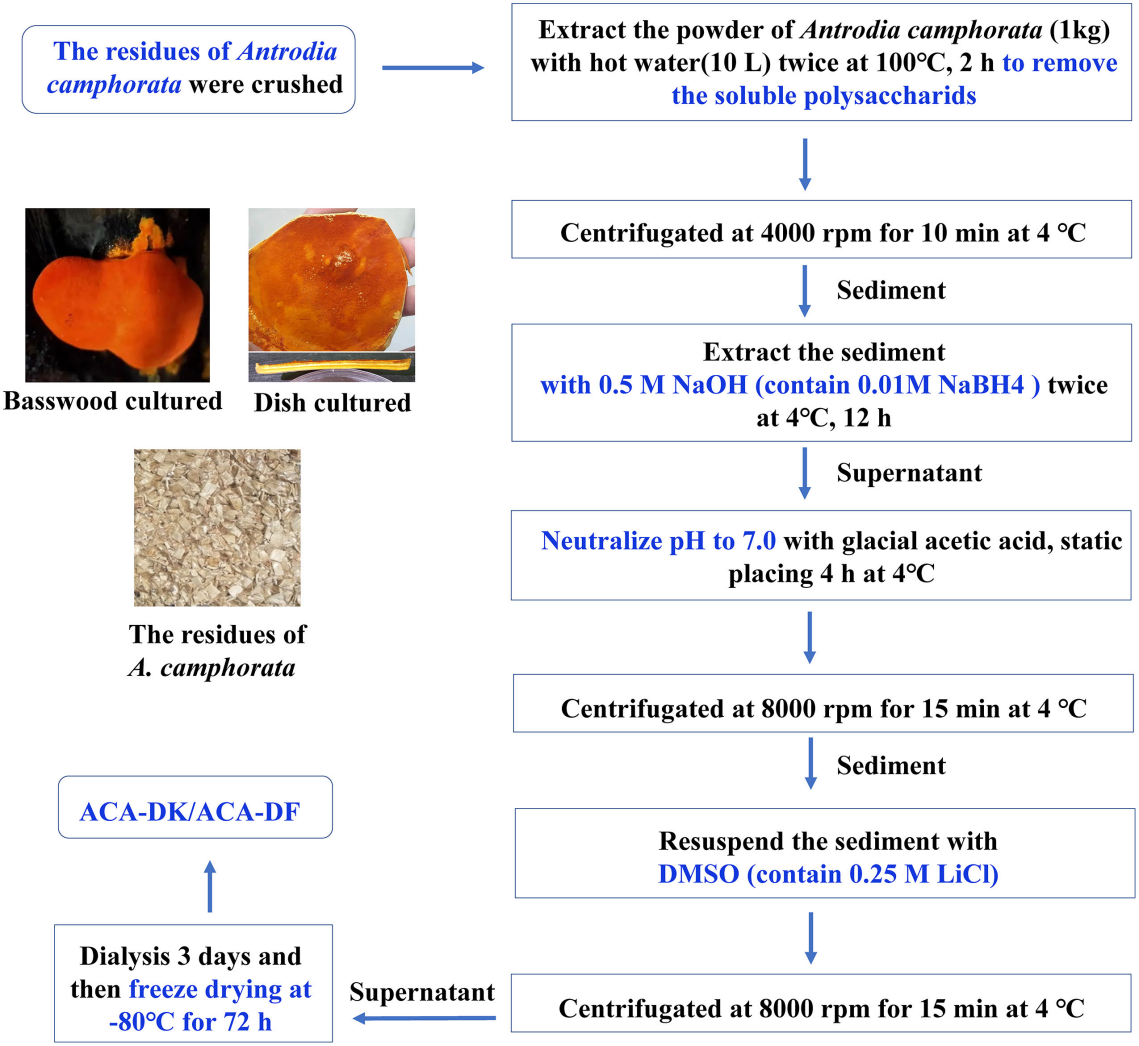
Flow chart representative of the extraction processes of dietary fibers ACA-DK and ACA-DF from the residues of *A. camphorata*.

The samples of *A. camphorata* from basswood-cultivated and dish-cultured extract used in this experiment were the residues of ethanol and hot water extraction. As shown in Figure 1, the dietary fiber ACA-DK (basswood cultivated) and ACA-DF (dish cultured) were extracted by NaOH-DMSO. As shown in Table 1, the extraction rates of ACA-DK and ACA-DF were 6.92 and 3.29%, respectively. The dietary fiber contents in these two samples reached 88.5 and 84.7%, respectively, and the protein contents were low (<3%). ACA-DK and ACA-DF did not contain uronic acid, indicating that these two dietary fibers are neutral sugars.

**Table 1:** Chemical composition and physicochemical properties of ACA-DK and ACA-DF.

**Component (%)**	**ACA-DK**	**ACA-DF**
Moisture	3.02 ± 0.37^a^	4.57 ± 0.24^b^
Protein	1.67 ± 0.22^a^	2.91 ± 0.32^b^
Total sugar	5.58 ± 0.32^a^	4.83 ± 0.26^b^
IDF	88.5 ± 0.25^a^	84.7 ± 0.34^b^
Extraction yield	6.92 ± 0.11^a^	3.34 ± 0.13^b^
Uronic acid	0	0

*Data are presented as mean values ± SD (n = 3). Results were expressed as %, content percentage from the ACA-DK and ACA-DF residues, and all the other data were measured with ACA-DK and ACA-DK as sample*.

*The significance of differences between the data was assessed using One-way ANOVA by Dunnett's tests, with the level of significance set at P < 0.05*.

The monosaccharide composition of dietary fiber has an important influence on its properties. As shown in Supplementary Figures 1A–C, both ACA-DK and ACA-DF contain four kinds of monosaccharides, without glucosamine and galactosamine. It was indicated that ACA-DK and ACA-DF were neutral dietary fiber, which were consistent with the previous results. The monosaccharide composition of ACA-DK and ACA-DF is similar, mainly glucose (65.44, 62.29%), xylose (13.35, 16.25%), mannitol (13.12, 13.15%), and fucosaccharide (6.22, 7.88%) (Table 2). Based on the solubility of these two dietary fibers, the gel chromatography was used to determine their molecular weight. As shown in Supplementary Figure 1D, the values of weight average molecular weight (Mw) and number average molecular weight (Mn) of ACA-DK were 18.28 and 10.04 kDa, respectively. The values of weight average molecular weight (Mw) and number average molecular weight (Mn) of ACA-DF were 15.35 and 10.39 kDa, respectively.

**Table 2:** Monosaccharide compositions of ACA-DK and ACA-DF.

**Samples**	**Monosaccharide compositions (%)**			
	**Fuc**	**Glu**	**Xyl**	**Man**
ACA-DK	6.22 ± 0.23^a^	65.44 ± 2.11^a^	13.35 ± 0.31^a^	13.85 ± 0.27^a^
ACA-DF	7.88 ± 0.40^b^	62.29 ± 0.35^a^	16.25 ± 0.15^b^	12.67 ± 0.33^b^

*Values are represented as mean ± SD (standard deviation)*.

*Fuc, fucosaccharide; Glu, glucose; Xyl, xylose; Man, mannose*.

*The significance of differences between the data was assessed using One-way ANOVA by Dunnett's tests, with the level of significance set at P < 0.05*.

### Structure Analysis

#### SEM Analysis of ACA-DK and ACA-DF

**Figure 2:**
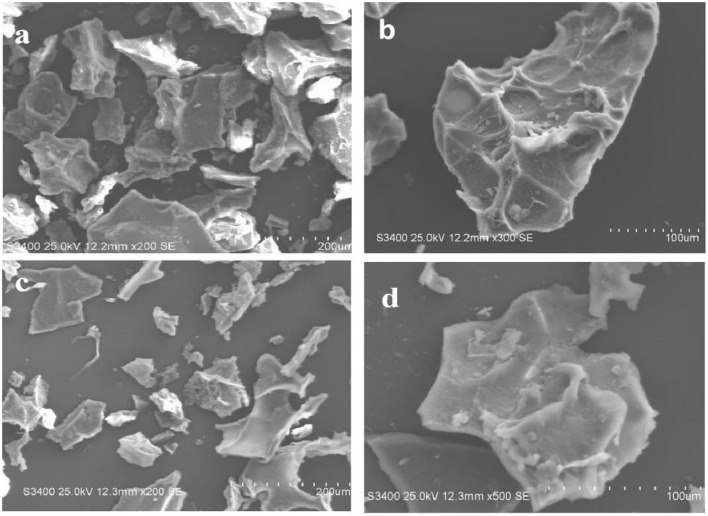
Microstructure of ACA-DK and ACA-DF observed by scanning electron micrographs (magnifications: 200X, 100X, respectively). **(a,b)** SEM image of ACA-DK; **(c,d)** SEM image of ACA-DF.

The morphology and structure of the ACA-DK and ACA-DF were analyzed by scanning electron microscope (SEM), and the results are shown in Figure 2. The micromorphologies of these two dietary fibers were similar, which showed irregular fragments with reticular lines on the surface. In addition, there are almost no spherical starch or protein particles on the surface of the two dietary fibers, which were consistent with the results of the previous composition determination.

#### FT-IR and X-Ray Analysis of ACA-DK and ACA-DF

**Figure 3:**
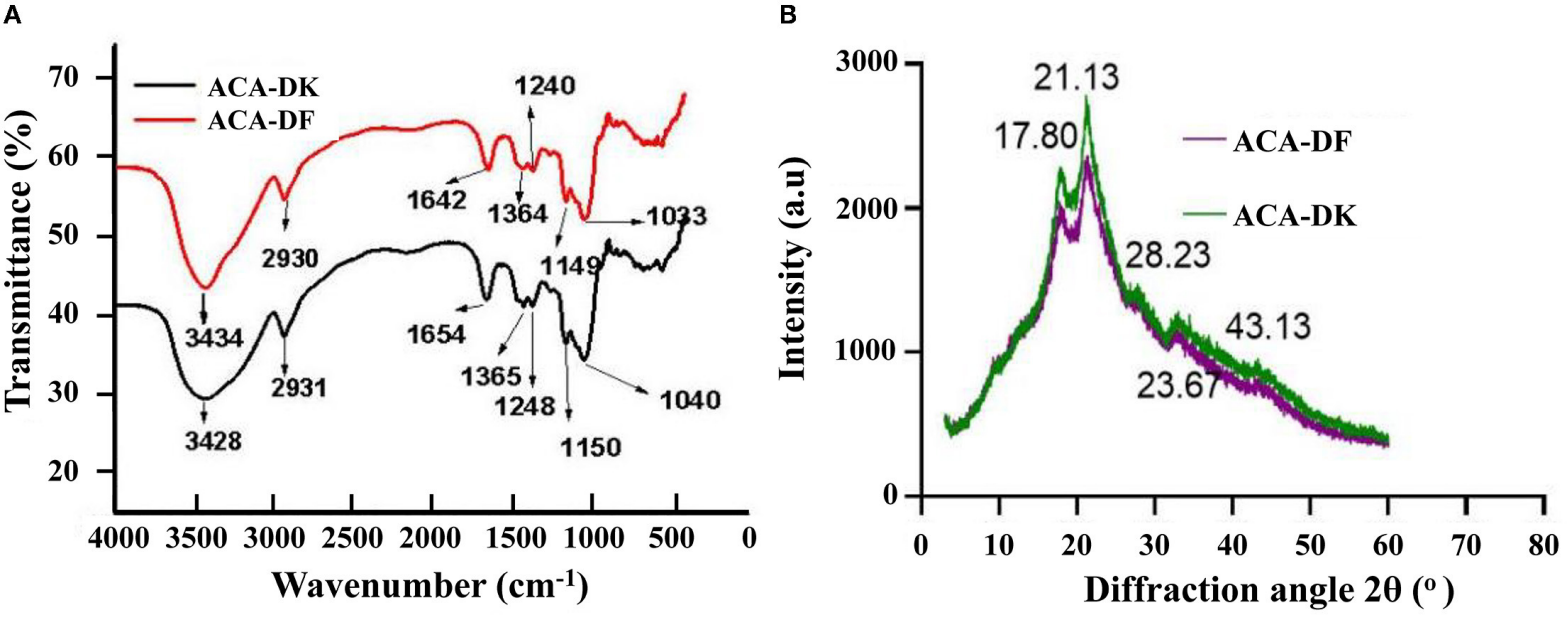
**(A)** FT-IR and **(B)** X-ray analysis of ACA-DK and ACA-DF.

The FT-IR spectroscopy of ACA-DK and ACA-DF was analyzed from 500 to 4,000 cm^−1^, which reflects functional groups of dietary fiber from *A. camphorata*. Similar typical peaks were observed from the spectral of ACA-DK and ACA-DF. As shown in Figure 3A, a strong absorption around 3,434 and 3,428 cm^−1^ for O-H group vibration, mainly of the native hemicelluloses (Sao et al., 1987). The notable bands around 2,930 and 2,931 cm^−1^ originated from C-H stretching of -CH_3_ or -CH_2_, indicating the presence of the typical structure of cellulose (Ma and Mu, 2016). The characteristic absorption band at 1,642 and 1,654 cm^−1^ was mostly attributed to the C=O stretching of carboxyl groups that are interconnected with cellulose chains by forming intermolecular hydrogen bonds (Qi et al., 2015). The minor peak at 1,364 and 1,365 cm^−1^ were mainly due to C–H bending vibrations related to the structure of hemicelluloses (Cherian et al., 2008), while the bands at 1,240 and 1,248 cm^−1^ were the O-H or C-O group vibrations in hemicelluloses (Bian et al., 2012). The prominent broad bands between 1,150 and 1,040 cm^−1^ originated from the acyl-oxygen (CO-OR) stretching vibration of hemicelluloses, indicating a dominant xylan of the alkali-soluble hemicelluloses (Peng and She, 2014). The results of FT-IR shown that the dietary fiber ACA-DK and ACA-DF had the typical functional groups of cellulose and xylan.

X-ray diffractometry was used to analysis the crystalline behavior and structure of ACA-DK and ACA-DF from *A. camphorata*. As shown in Figure 3B, the X-ray diffraction patterns of ACA-DK and ACA-DF were similar in shape. The distinct diffraction peaks were observed at 2θ = 17.80° and 21.13°, which represents typical crystalline cellulose I region and amorphous area (Qi et al., 2015; Karaman et al., 2017). The characteristics of these two peaks are similar to the dietary fiber previously reported (Qi et al., 2015; Hua et al., 2019). The observed irregular minor peaks from 28.23° to 43.13° might be due to the denaturing effect of alkaline solutions on cellulose (Ma and Mu, 2016). The crystallinity indices of ACA-DK and ACA-DF were 37.21 and 34.23%, which were close to the 36.60% crystallinity index of okara and 34.24% of ginseng (Ullah et al., 2017; Hua et al., 2019).

#### *In vitro* Adsorption Capacity of ACA-DK and ACA-DF

**Figure 4:**
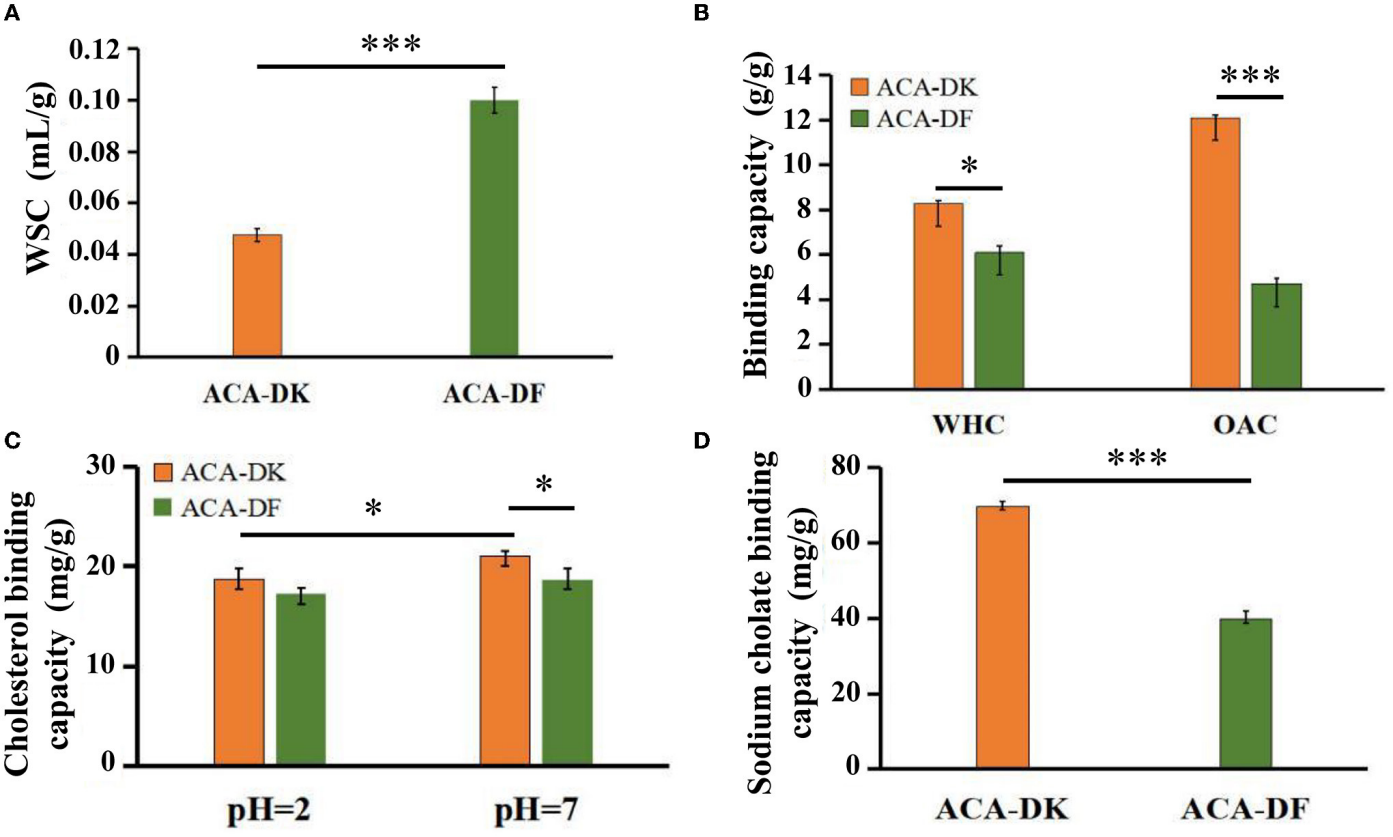
Physicochemical and functional properties of ACA-DK and ACA-DF. **(A)** Water swelling capacity (WSC); **(B)** water holding capacity (WHC) and oil binding capacity (OBC); **(C)** cholesterol binding capacity (CBC) at different pH; **(D)** sodium cholate binding capacity (SCBC). Results of experiments are express as mean ± SEM for each experimental group (*n* = 3). Significant differences were compared with each two groups (*P < 0.05, ***P < 0.001).

The physicochemical properties of ACA-DK and ACA-DF, including water swelling capacity (WSC), water holding capacity (WHC), and oil adsorption capacity (OAC) were presented and compared in Figures 4A,B. There were significant differences between ACA-DK and ACA-DF in terms of WSC characteristics. The WSC of ACA-DF (0.98 mL/g) is twice as much as ACA-DK (0.05 mL/g). The WHC values of ACA-DK and ACA-DF were 8.28 and 6.09 g/g, respectively, which showed better water holding capacity than dietary fiber from foxtail millet and bamboo shell (Luo et al., 2017; Zhu et al., 2018). As shown in Figure 4B, the OAC of ACA-DK (12.09 mg/g) was 2.6 times higher than ACA-DF (4.70 mg/g). ACA-DK and ACA-DF cholesterol binding capacities (CBC) at pH 2.0 and pH 7.0 were measured and are given in Figure 4C. The CBC of ACA-DK at pH 7.0 (20.99 mg/g) was higher than that of ACA-DF (18.02 mg/g) significantly. There is no significant difference at pH 2.0. As shown in Figure 4D, ACA-DK showed superior sodium cholate binding capacity (SCBC) (69.68 mg/g) than that of ACA-DF.

#### Effect of ACA-DK and ACA-DF on RAW 264.7 Cells

**Figure 5:**
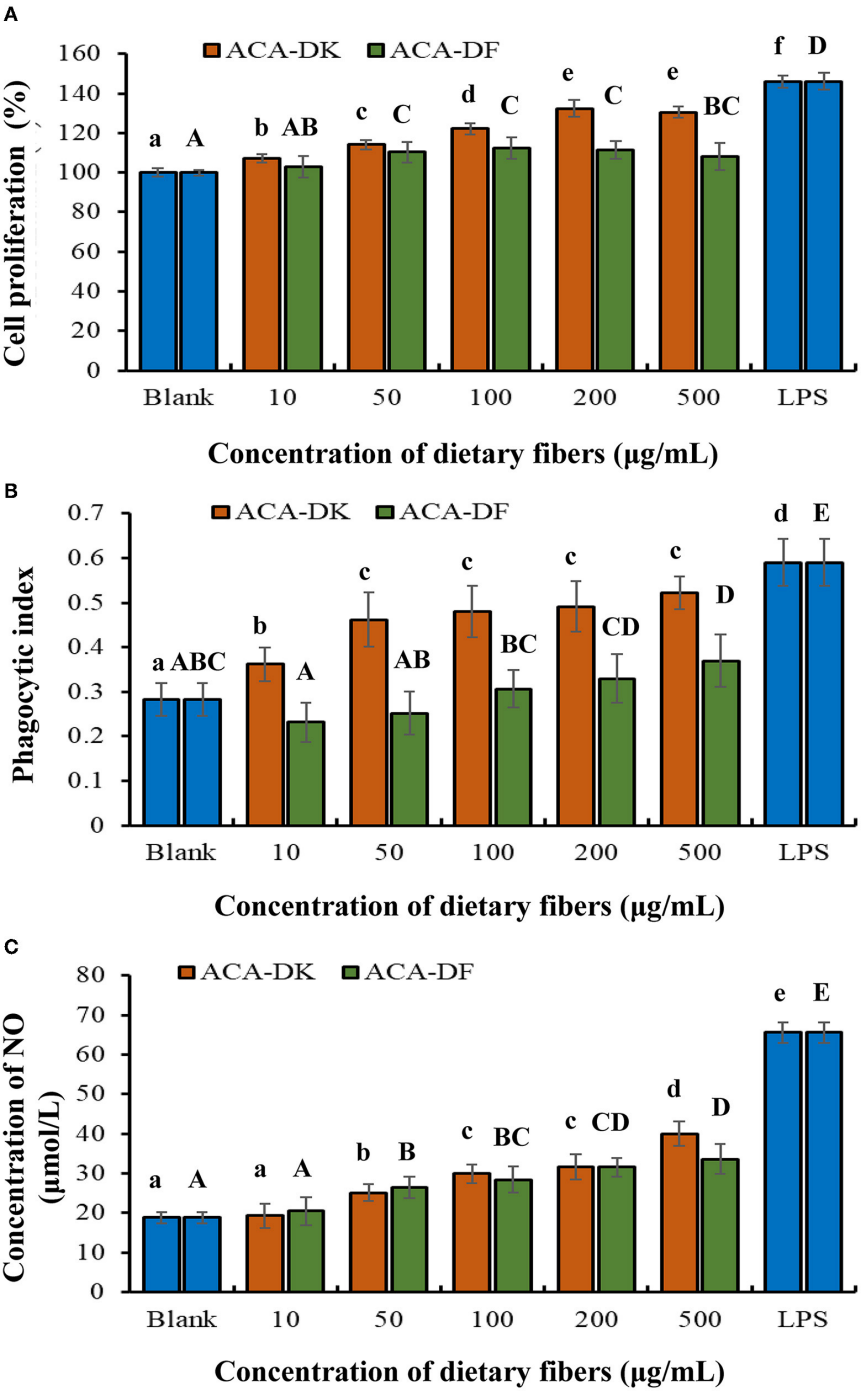
Effect of ACA-DK and ACA-DF on the **(A)** proliferation, **(B)** phagocytic activity, and **(C)** NO production of RAW 264.7 cells. The cells were treated with increasing concentrations of ACA-DK and ACA-DF (10, 50, 100, 200, 500 μg/mL) for 24 h, respectively; LPS (1μg/mL) as a positive control. Results of experiments are express as mean ± SEM for each experimental group (*n* = 6). The significance of differences between the data was assessed using one-way ANOVA by Dunnett's tests, with the level of significance set at *P* < 0.05.

To analyze whether ACA-DK and ACA-DF cause toxicity on RAW 264.7 macrophages, the cell viability was measured by MTT assay. As shown in Figure 5A, compared with the blank group, the two dietary fiber sample (10, 50, 100, 200, 500 μg/mL) treatment groups had significantly increased proliferation of RAW 264.7 macrophages after 24 h of cultured (*P* < 0.05). While the cell viability of ACA-DK was significantly higher than that of ACA-DF, when the concentration was up to 100 μg/mL. Neutral red uptake was carried out to explore the effects of ACA-DK and ACA-DF on the phagocytic activity of LPS-treated RAW 264.7 macrophages cells. As shown in Figure 5B, RAW 264.7 cells in the LPS treatment group showed a dramatic increase in the level of phagocytic activity as compared with the blank group (*P* < 0.01). The two dietary fiber samples could both enhance the phagocytic ability of RAW 264.7 cell significantly. However, the phagocytic uptake ability of the RAW 264.7 cells was promoted significantly and presented in a good concentration-dependent manner after treated with ACA-DK. It was noted that the phagocytic ability of RAW 264.7 cells with a high concentration of ACA-DK was significantly lower than that of the LPS-treated group. The results suggest that ACA-DK is superior to ACA-DF in the activation of phagocytosis.

Effects of ACA-DK and ACA-DF on NO production are presented in Figure 5C. A significant increase in NO production was observed in the LPS-treated group when compared with the blank (*P* < 0.01). The production of NO was significantly promoted by ACA-DK and ACA-DF in a time- and concentration-dependent manner (*P* < 0.01), and the two dietary fiber had the similar ability of promoting NO production.

#### Effect of ACA-DK and ACA-DF on TNF-α and IL-1β Secretion

**Figure 6:**
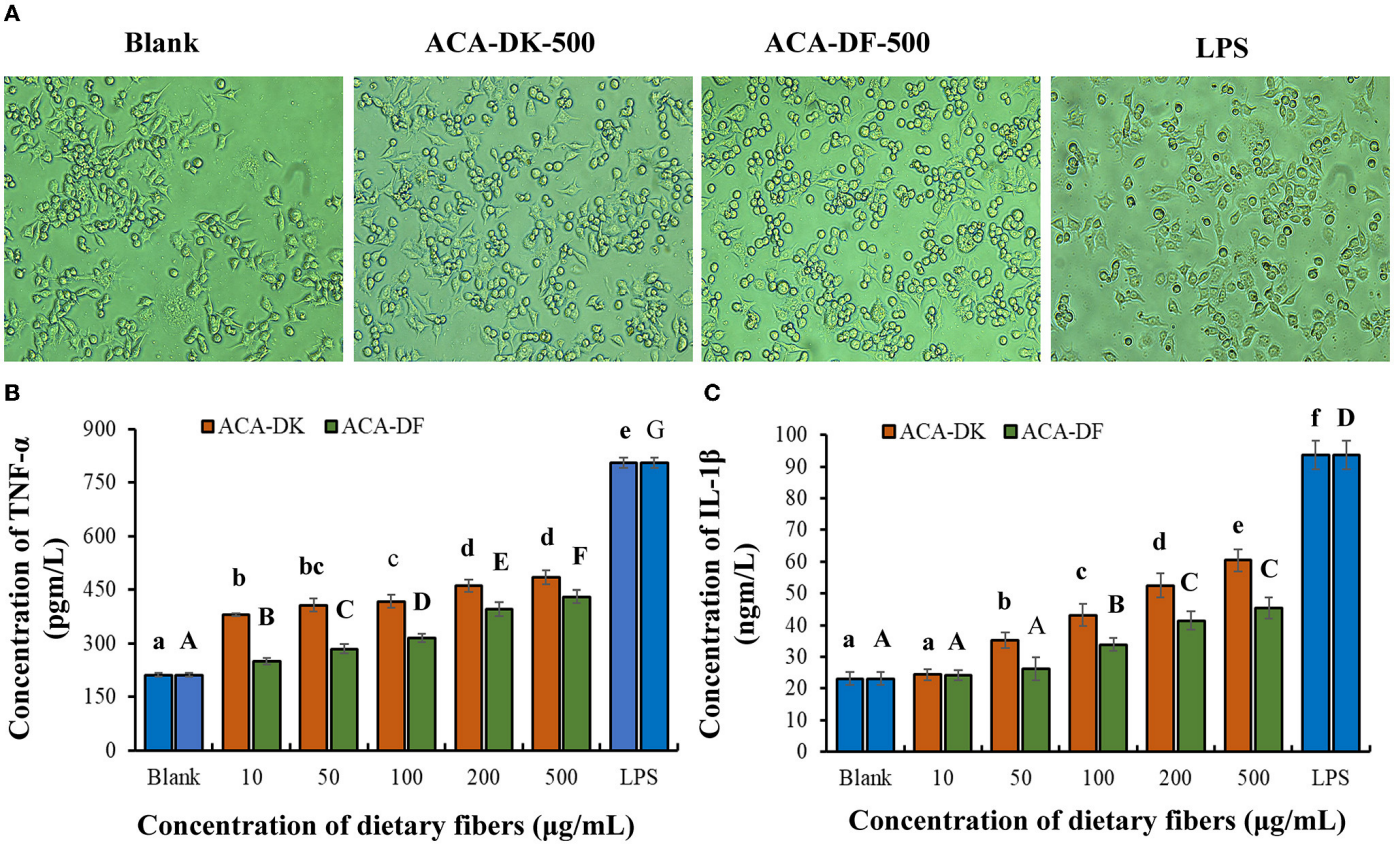
Effect of ACA-DK and ACA-DF on the **(A)** cell morphology, **(B)** TNF-α, and **(C)** IL-1β production of RAW 264.7 cells. The cells were treated with increasing concentrations of ACA-DK and ACA-DF (10, 50, 100, 200, 500 μg/mL) for 24 h, respectively; LPS (1 μg/mL) as a positive control. Results of experiments are express as mean ± SEM for each experimental group (*n* = 6). The significance of differences between the data was assessed using one-way ANOVA by Dunnett's tests, with the level of significance set at *P* < 0.05.

The effects of each group on RAW 264.7 cell morphology was observed under an inverted microscope. As shown in Figure 6A, the morphology of RAW 264.7 cells activated by LPS was almost completely changed, with pseudopodia protruding and spindle-shaped, and the deformation was greater than that of the blank group. After treatment with ACA-DK and ACA-DF, some RAW 264.7 cells were stretched, extended pseudopods, or spindle-shaped compared with normal cells. The change of RAW 264.7 cells morphology could increase the contact area with the outside material and facilitate phagocytosis and absorption.

To further investigate the immunomodulatory effects of ACA-DK and ACA-DF on macrophage cells, pro-inflammatory cytokines, such as TNF-α and IL-1β, were determined using ELISA kits. As shown in Figures 6B,C, stimulation with LPS resulted in a significant increase in the production of pro-inflammatory cytokines of TNF-α and IL-1β compared with the blank group (*P* < 0.01). Stimulatory activity of ACA-DK and ACA-DF on macrophages for 24 h caused an increase in the production of TNF-α and IL-1β cytokines in a dose-dependent manner. ACA-DK could significantly promote the release of TNF-α and IL-1β cytokines (*P* < 0.01) compared to ACA-DF in the concentration range of 50–500 μg/mL. After 24 h of the ACA-DK treatment, the secretion of TNF-α and IL-1β reached the highest levels for 484.43 ± 18.77 pg/mL and 60.39 ± 3.47 ng/mL, respectively.

## Discussion

Dietary fiber is widely found in grains, fruits, mushrooms, and other foods and has good biological activity. Although dietary fiber is hardly degraded in the intestine, its good adsorption and looseness could promote intestinal peristalsis and promote the excretion of dietary cholesterol and fat from the body. *Antrodia camphorata* is a medicinal fungus with a variety of bioactivities. At present, most of *A. camphorata* is treated with solvents to extract small molecular bioactive components and polysaccharides. However, the residue after extraction has not been effectively developed, and the waste of resources is more serious. In this study, the dietary fiber of *A. camphorata* residue was obtained by alkaline extraction, and its adsorption properties and immune regulation properties were studied.

The dietary fiber extracted from residue of *A. camphorata* is composed of mainly cellulose and xylan. There are significant differences in the structure of the dietary fiber extract from common foods. The monosaccharide composition of ACA-DK and ACA-DF is significantly different from dietary fiber derived from grains and fruits (Dong et al., 2019; Hua et al., 2019). The ACA-DK and ACA-DF have lower molecular weights, which may be related to the alkaline extraction process. The results of SEM showed that ACA-DK and ACA-DF had a loose and irregular block structure. This irregular structure is similar to the dietary fiber from millet bran and *Sagittaria sagittifolia L* (Dong et al., 2019; Gua et al., 2020). This loose structure has a great influence on its physical and chemical properties, and could improve its ability to absorb water, oil, and salt (Chen et al., 2013). The fractures of ACA-DK and ACA-DF are irregular, which may be caused by the destruction of the fiber matrix due to alkaline extraction (Khawas and Deka, 2016). At the same time, alkaline extraction also led to the exposure of non-polar groups of dietary fat, the smaller particle size, and the formation of a large number of protrusions on the surface, which significantly increased the specific surface area of dietary fat (Zhu et al., 2015; Wang et al., 2019). This structural characteristic enables *A. camphorata* dietary fibers to have a strong binding capacity for substances such as oil and cholesterol, thereby avoiding the intake of excessive dietary oil and cholesterol.

To evaluate the crystallinity index, a non-linear multi-peak fitting function was used for peak separation of each XRD (Cleven et al., 1978). The intensity of ACA-DK is higher than that of ACA-DF, indicating that the DMSO/LiCl treatment disintegrated the crystalline and amorphous areas of dietary fiber (Ullah et al., 2017). The DMSO/LiCl solvent system could disrupt the hydrogen bonds between cellulose molecules irreversibly, so as to effectively separate and extract water-insoluble components in *A. camphorata* cell walls, such as cellulose and hemicellulose (Wang et al., 2013).

The adsorption capacity of dietary fiber plays a very important role in its function. The WSC of *A. camphorata* dietary fiber were similar to tomato peel fiber and lower than carrot dietary fibers (González et al., 2011; Yang et al., 2021). This may be due to the fact that the dietary fibers of *A. camphorata* are mainly composed of cellulose and xylan, so the swelling ability is weak when mixed with water (Lecumberri et al., 2007). WHC is an important indicator to evaluate the function of dietary fiber, and it could change the texture of food (Li et al., 2013; Liu et al., 2021). The WHC of ACA-DK and ACA-DF were similar to that of rice bran dietary fiber (Zhao et al., 2018). Additionally, the higher WHC of ACA-DK is associated with the porous matrix structure with hydrophilic sites, which could hold large amounts of water through hydrogen bonds (Margareta and Nyman, 2002).

Although dietary fiber not digested by endogenous enzymes within the human intestinal tract, the adsorption capacity of fiber to retain oil, cholesterol and bile salts were important for food applications (Zhu et al., 2018). The OAC, SCBC, and CBC of dietary fiber are commonly used as indicators of lipophilicity (Zhu et al., 2018). The CBC of dietary fiber, which has been shown to reduce serum cholesterol levels and the risk of cardiovascular disease (Nsor-Atindana et al., 2012). In this study, the OAC of ACA-DK was found to be better than that reported for dietary fibers from some vegetables, fruits, and grains (Yang et al., 2021; Zhao et al., 2021) and similar to that of cocoa dietary fiber (Nsor-Atindana et al., 2012). The CBC of ACA-DK was similar to the behavior of grapefruit dietary fiber (Wang et al., 2015) and much better than foxtail millet bran (Zhu et al., 2018). The pH value has a significant impact on the adsorption ability of dietary fiber to cholesterol. For ACA-DK, the binding ability of cholesterol at pH 7.0 is higher than that at pH 2.0 significantly. The result indirectly demonstrated that the CBC of ACA-DK in the small intestine was higher than that in the stomach. The SCBC of ACA-DK was close to that of ginseng dietary fiber (Hua et al., 2019) but higher than that of the dietary fiber isolated from foxtail millet bran and bamboo shoot shell (Luo et al., 2017; Dong et al., 2019).

According to Femenia et al. (1997), oil binding capacity depends on the surface properties of dietary fibers, but not on charge density and the hydrophobic nature of the particles. However, other studies have shown that the total charge density and hydrophobicity of dietary fibers may affect the OBC (Figuerola et al., 2005; Gomez-Ordonez et al., 2010). For similar surface properties, the higher content of cellulose for ACA-DK may be the reason why its binding capacity for oil, cholesterol, and bile salts is higher than that of ACA-DF. It indicated that ACA-DK has potential to be used as an ingredient in dietary fiber-rich food stuffs requiring oil retention and reducing oil absorption during digestion processing in the human. Also it could inhibit the absorption of cholesterol and bile acid in the intestinal tract and promote its elimination.

Biomacromolecules such as polysaccharides and glycopeptides/glycoproteins could activate immune cells to play a role in immunomodulatory. In the intestinal tract, as a biomacromolecules, dietary fiber has a good ability to bind fat and cholesterol and reduce chronic inflammation. In order to further investigate whether the dietary fiber of *A. camphorata* has a similar function, the immune regulation properties of ACA-DK and ACA-DF on RAW 264.7 cells were investigated. Macrophages are an important in the body's immune system and play crucial roles in both adaptive and innate immunities. Studies have shown that NO has a wide range of immunological significance and plays a key role in the immune system as an important signal transduction medium (Rahat and Hemmerlein, 2013). When macrophages are activated, NO produced has a major cytotoxic effect on nonspecific immunity (Nie et al., 2018). The cytokines (TNF-α and IL-1β) play an important role in inflammatory reactions and immune responses (Cheng et al., 2019). The results of ACA-DK were similar to those of the previously reported potato- and green gram alkali-extracted polysaccharides (Ketha and Gudipati, 2018; Tang et al., 2019). The ACA-DK could promote the release of TNF-α and IL-1β cytokines in macrophages more effectively than ACA-DF. Moreover, ACA-DK was less able to promote cytokine secretion than the LPS group.

## Conclusion

In this study, the structural and functional properties of dietary fibers in *A. camphorata* were investigated. The dietary fiber ACA-DK extract from residues of *A. camphorata* by using the NaOH-DMSO method has the structural characteristics of cellulose, with high purity, lower molecular weight, and appearance of irregular fragments. ACA-DK has good ability *in vitro* adsorption, especially for oil, cholesterol, and sodium cholate. Cell experiments show that ACA-DK could promote the proliferation of macrophages RAW 264.7, activate the synthesize of NO, phagocytosis, and other immune capabilities. The edible fungus *A. camphorata* is a good source of functional dietary fiber. The study on *A. camphorata* dietary fiber is of great significance for the comprehensive utilization of *A. camphorata* resources. In future, further evaluation of its cholesterol-lowering activity using animal experiments could lay a foundation for the functional expansion of *A. camphorata* dietary fiber.

## Data Availability Statement

The original contributions presented in the study are included in the article/Supplementary Material, further inquiries can be directed to the corresponding author.

## Author Contributions

YX: experiment, investigation, data curation, and writing—original draft. PM and LL: experiment, resources, and methodology. SL, ZT, and XY: experiment and statistical analysis. FX, HZ, and GW: conceptualization, software, and assisted the statistical analysis. ZX, JL, and LA: grammar and revision of the manuscript. All authors contributed to the article and approved the submitted version.

## Funding

This work was supported by the Natural Science Foundation of China (Grant Number 31871757), the Shanghai Agriculture Applied Technology Development Program (Grant Number 2019-02-08-00-07-F01152), and the Shanghai Engineering Research Center of Food Microbiology Program (Grant Number 19DZ2281100).

## Conflict of Interest

JL was employed by Honest and Humble Biotechnology Co., Ltd. The remaining authors declare that the research was conducted in the absence of any commercial or financial relationships that could be construed as a potential conflict of interest.

## Publisher's Note

All claims expressed in this article are solely those of the authors and do not necessarily represent those of their affiliated organizations, or those of the publisher, the editors and the reviewers. Any product that may be evaluated in this article, or claim that may be made by its manufacturer, is not guaranteed or endorsed by the publisher.
