# Supplementary material for: Structural and Potential Functional Properties of Alkali-Extracted Dietary Fiber From Antrodia camphorata
Source: Front Microbiol. 2022 Jul 7;13:921164. doi: 10.3389/fmicb.2022.921164 (PMC9301256; doi:10.3389/fmicb.2022.921164)
Supplement: Supplementary file 1 [file Table_1.DOCX]

Supplementary Material


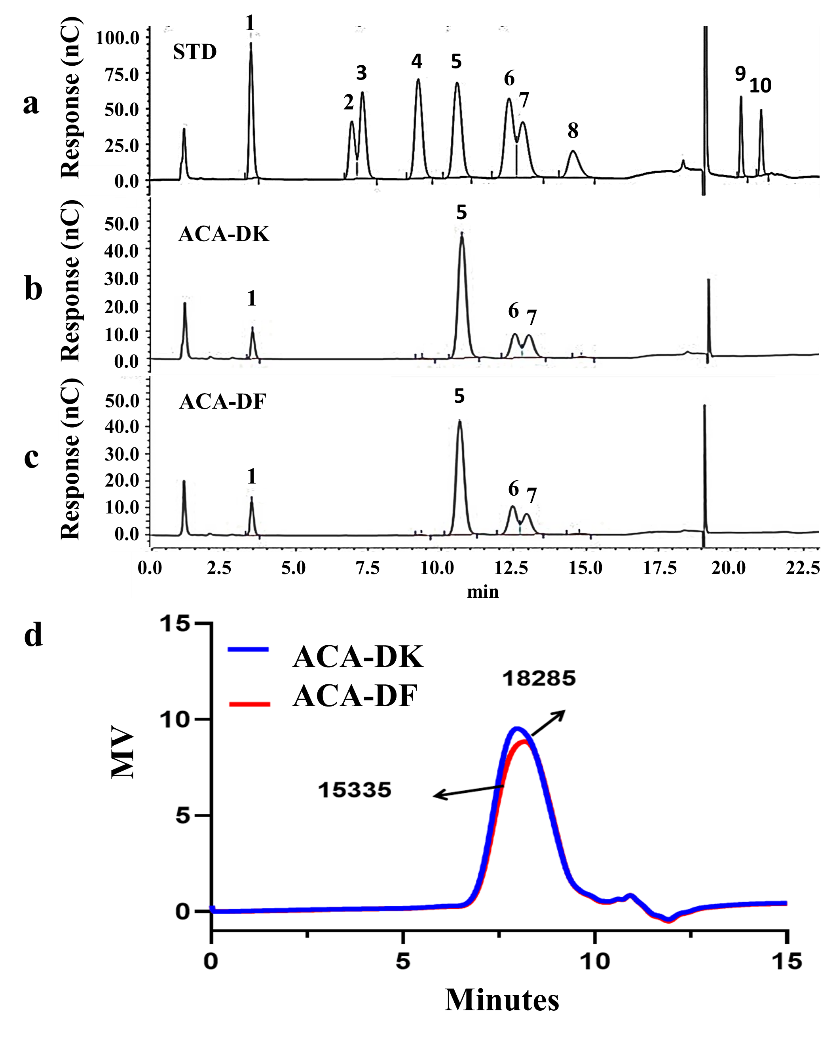


**Supplementary Figure 1.** The monosaccharide composition and molecular weights of ACA-DK and ACA-DF. A: The monosaccharide standards; peaks: 1: Fucose; 2: Rhamnose; 3: Arabinose; 4: Galactose; 5: Glucose; 6: Xylose; 7: Mannose; 8: Fructose; 9: Galacturonic acid; 10: Glucuronic acid; B: The monosaccharide compositions of ACA-DK; C: The monosaccharide compositions of ACA-DF; D: The molecular weights of ACA-DK and ACA-DF.
